# Comparison of deflection forces of esthetic archwires combined with ceramic brackets*

**DOI:** 10.1590/1678-7757-2017-0220

**Published:** 2018-01-24

**Authors:** Murilo MATIAS, Marcos Roberto de FREITAS, Karina Maria Salvatore de FREITAS, Guilherme JANSON, Rodrigo Hitoshi HIGA, Manoela Fávaro FRANCISCONI

**Affiliations:** 1 Universidade de São Paulo, Faculdade de Odontologia de Bauru, Departamento de Odontopediatria, Ortodontia e Saúde Coletiva, Bauru, São Paulo, Brasil.; 2 Faculdade Ingá, Departamento de Ortodontia, Maringá, Paraná, Brasil.

**Keywords:** Orthodontic archwires, Orthodontic brackets, Esthetics, Coating

## Abstract

Coated archwires and ceramic brackets have been developed to improve facial esthetics during orthodontic treatment. However, their mechanical behavior has been shown to be different from metallic archwires and brackets. Therefore, the aim of this study was to compare the deflection forces in coated nickel-titanium (NiTi) and esthetic archwires combined with ceramic brackets.

**Material and Methods:**

Non-coated NiTi (NC), rhodium coated NiTi (RC), teflon coated NiTi (TC), epoxy coated NiTi (EC), fiber-reinforced polymer (FRP), and the three different conventional brackets metal-insert polycrystalline ceramic (MI-PC), polycrystalline ceramic (PC) and monocrystalline ceramic (MC) were used. The specimens were set up on a clinical simulation device and evaluated in a Universal Testing Machine (Instron). An acrylic device, representative of the right maxillary central incisor was buccolingually activated and the unloading forces generated were recorded at 3, 2, 1 and 0.5 mm. The speed of the testing machine was 2 mm/min. ANOVA and Tukey tests were used to compare the different archwires and brackets.

**Results:**

The brackets presented the following decreasing force ranking: monocrystalline, polycrystalline and polycrystalline metal-insert. The decreasing force ranking of the archwires was: rhodium coated NiTi (RC), non-coated NiTi (NC), teflon coated NiTi (TC), epoxy coated NiTi (EC) and fiber-reinforced polymer (FRP). At 3 mm of unloading the FRP archwire had a plastic deformation and produced an extremely low force in 2; 1 and 0.5 mm of unloading.

**Conclusion:**

Combinations of the evaluated archwires and brackets will produce a force ranking proportional to the combination of their individual force rankings.

## Introduction

In modern society, the esthetic aspect of orthodontic appliances is important, particularly because more adult patients are seeking for orthodontic care^2^. The availability of different appliances, such as lingual orthodontics, clear aligners, and esthetic labial fixed appliances are well-accepted solutions by these patients who demand a high esthetic treatment^30^ ^,^ ^31^. Brackets and archwires are the two main groups of materials used in orthodontic treatment. The use of esthetic orthodontic archwires in association with esthetic brackets is likely the next step to enhance the esthetics of orthodontic appliances^28^.

Although esthetics are desired by patients and orthodontists, proper and efficient function of the appliance is mandatory^22^. In the case of brackets, the introduction of composite and ceramic brackets solve the problem^27^. Ceramic brackets are available in two types; conventional and with metal-insert. The latter produces less frictional forces against conventional (uncoated) archwires^6^. Regarding archwires, a number of alternatives have been explored to create esthetic archwires that would allow efficient orthodontic treatment. Metal archwires, particularly nickel-titanium (NiTi) alloys, have been coated with either tooth-colored polymers or inorganic materials. Although these archwires might be considered more esthetic, a number of problems have been identified. An esthetic archwire lacks translucency and ideal transparency. Furthermore, the outer coating can wear out or peel, and the bending of the archwire is limited^24^.

The materials traditionally used to coat archwires are synthetic fluoropolymers, such as polytetrafluoroethylene (PTFE), epoxy resins or a combination of them. Disadvantages in durability and surface properties have been reported, such as tearing and color changing of these coatings in clinical conditions. Since esthetic archwires have shown nearly the same level of biocompatibility as metallic wires, their clinical use may be considered safe^26^. Efforts have been made to investigate and develop fiber-reinforced composite archwires suitable for use in clinical orthodontics, but commercial availability has been slowly progressing^4^ ^,^ ^5^ ^,^ ^15^.

During the coating application process on the archwire, a previous heat treatment is needed on its surface to produce an effective adhesion of the coating layer. As a result, the mechanical properties of metallic archwires could be affected during this process.

The mechanical properties of orthodontic archwires can be assessed by a 3-point bending test or a clinical simulation device, which evaluates the load-deflection properties, considered the most important parameters to determine the biologic nature of tooth movement. Considering the difficulty to directly evaluate periodontal ligament stresses, the only way to estimate these parameters is by knowing the magnitude of forces applied to the teeth. Thereby, *in vitro* studies try to aid orthodontists to design and select an orthodontic mechanics that is not only efficient and biologically safe, but also esthetic pleasant to patients.

The aim of this study was to compare the load-deflection properties of coated nickel-titanium (NiTi) and esthetic archwires combined with conventional ceramic brackets, by using a clinical simulation device.

## Material and methods

### Material

**Figure 1:**
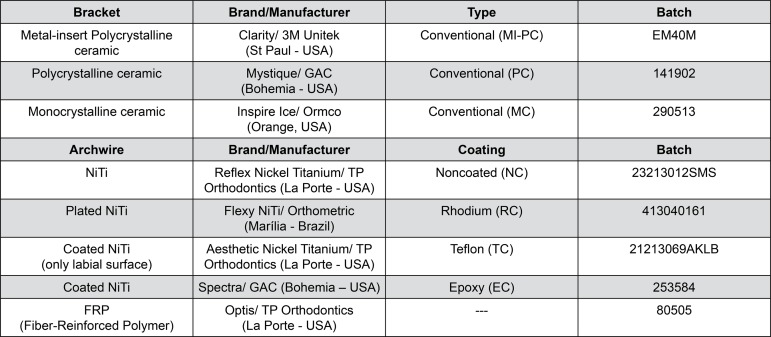
Experimental groups of brackets (0.022x0.028-in) and archwires (0.016-in)

Three clinical simulation devices were used in this study. Each of them received a different type of conventional esthetic bracket, varying according to its composition. All brackets had 0.022x0.028-inch slot size and were ligated by elastomeric ligatures (Super Slick^®^ Mini Stix Ligature Ties, TP Orthodontics; La Porte, Indiana, USA) with outer diameter of 3.23 mm in the conventional way (“O” shaped). In these devices, four different NiTi archwires (with and without esthetic coating) and one purely esthetic archwire, with superelastic and mechanical properties similar to NiTi archwire, manufactured with a reinforced polymeric composite of plastic resin and fiberglass, were used (Figure 1).

The archwires, brackets and elastomeric ligatures used were from the same batch. All evaluated archwires were round with 0.016-inch diameter and had the same format. The specimens were divided into 15 groups using 10 archwires *per* group, totaling 150 tests.

### Methods

In order to internationally standardize the tests as adequately as possible, this study followed the ISO 15841 standard^16^.

**Figure 2:**
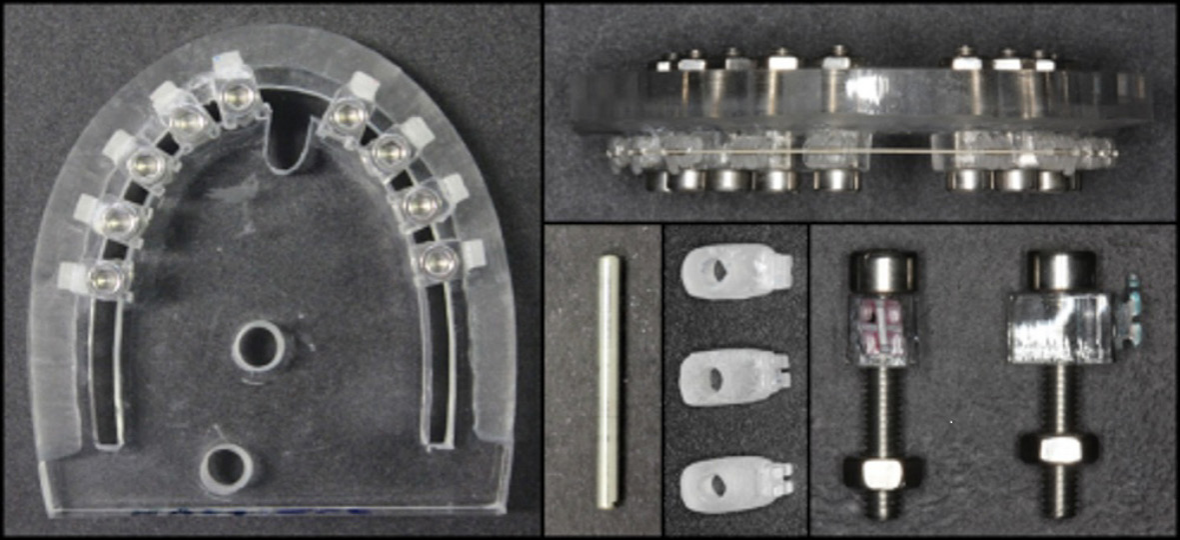
Acrylic resin plate with the structures in position and brackets bonded; acrylic device representative of the right maxillary central incisor and cylindrical metal structure

Archwire deflection was performed by a clinical simulation device representing the teeth of the maxillary arch, consisting of an acrylic resin plate with parabola shape, where structures that represent the maxillary teeth were affixed^10^ (Figure 2).

The brackets were bonded with cyanoacrylate ester gel (Super Bonder, Loctite, São Paulo, SP, Brazil) on acrylic structures and positioned so that the mesiodistal slot axes were aligned in the same vertical level, by using a 0.021x0.025-inch archwire. These structures were fixed with threaded screws in the bottom of the acrylic resin plate.

**Figure 3:**
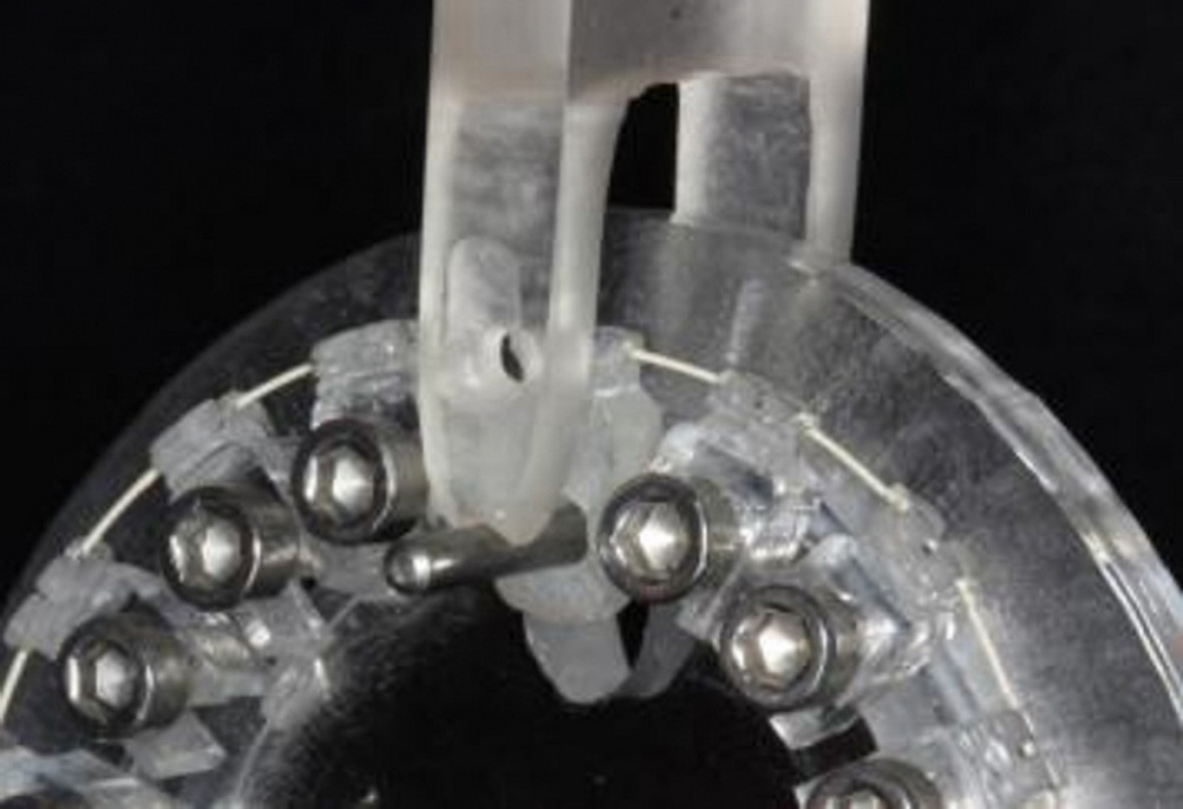
Tip of the universal testing machine applying a bucco-lingual pressure to the acrylic structure

The tests were performed on the structure corresponding to the maxillary right central incisor. Unlike the others, this structure was loose, enabling its bucco-lingual movement. It had a perforation that allowed a metal cylinder to be placed inside it for the activations. The tip of the activation device, attached to the testing machine had a rounded cut to fit the metal cylinder (Figure 3).

The inter-bracket distance was kept constant at 6 mm^29^, since the relation force/deflection is dependent, among other things, on this distance. The speed of the testing machine for the deflection was 2 mm/min.

Records of the force released by the wire deflection were made in 3; 2; 1 and 0.5 mm. The deflection of the wire attached to the bracket clinically corresponds to the beginning of the treatment, when the teeth are poorly positioned and the wire is forced into the accessory slots. Depending on the degree of crowding, teeth experience more or less force to allow proper alignment.

The deflection tests were performed with the Instron 3342 Universal Testing Machine with a load cell of 10 N. This load cell has an accuracy of 0.5% of the reading value when at 25°C. The load cell was maintained at this temperature and, therefore, the results had significant accuracy. Also, according to the ISO standard, the tests were always performed at the same temperature of 36±1°C for all test groups.

**Figure 4:**
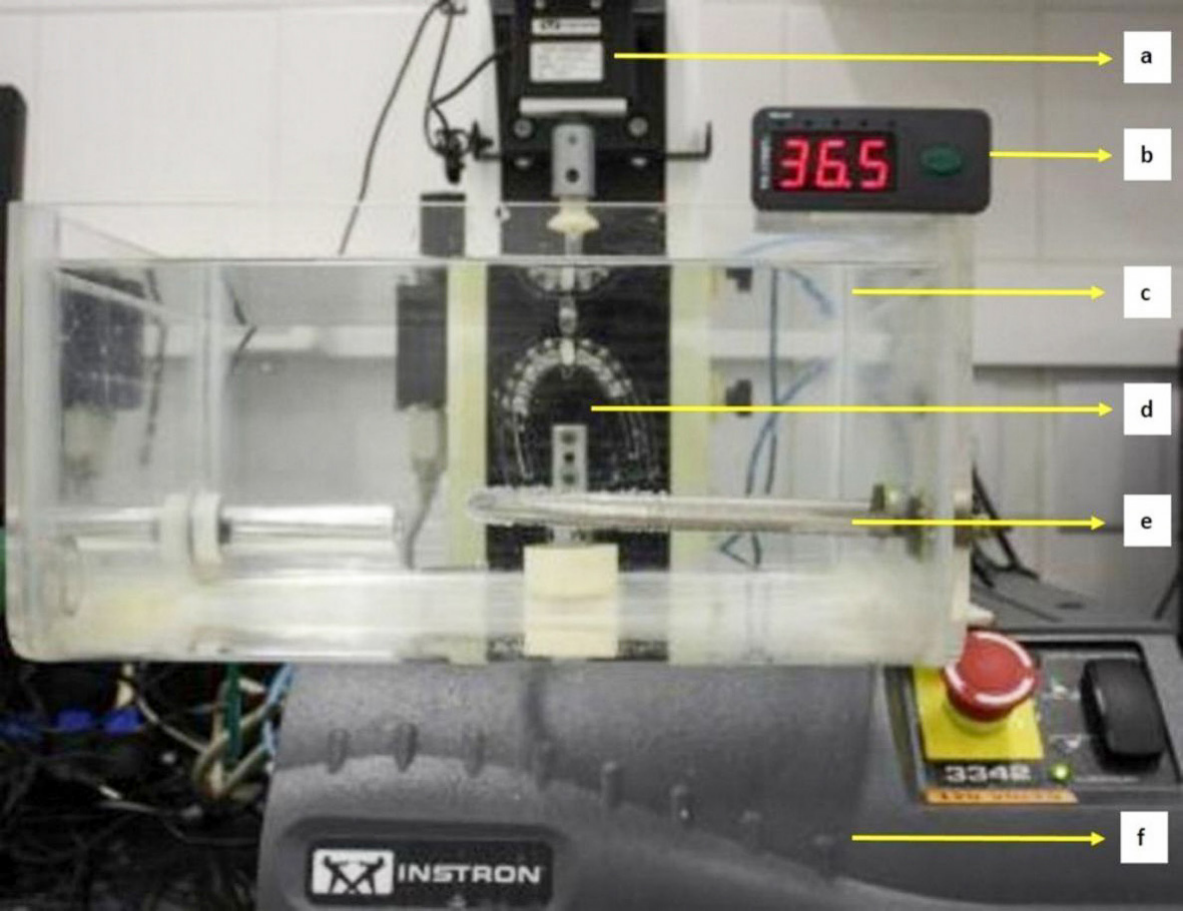
Clinical simulation device. (a- Load cell of 10N; b- Digital thermostat; c- Acrylic container; d- Acrylic resin plate; e- Submersible electric resistance; f- Universal Testing Machine)

An acrylic container with water at 36±1°C, maintained with the aid of submersible electric resistance, connected to a digital thermostat (TIC 17RGTi/9 model, Full Gauge Controls, Canoas, RS, Brazil), previously scheduled to stay in the desired temperature range, was adapted to the test machine^23^ ^,^ ^29^ (Figure 4). Before each test, the load cell was calibrated with the Bluehill Lite software (v.2.25, 2005).

### Statistical analyses

The sample size was calculated based on the ISO 15841 standard, which recommends six specimens for each group. However, to minimize the chances of any technical error and to increase the results reliability, a number of ten specimens were used for each group.

Normal distribution of the variables was evaluated with Kolmogorov-Smirnov tests. Because all variables showed normal distribution, parametric tests were used.

One-way Anova and Tukey tests were used to compare the forces delivered by the different archwires in different brackets and to compare the forces produced by different brackets used with different archwires.

All statistical analyses were performed with Statistica software (Statistica for Windows – Release 7.0, Copyright Statsoft, Inc. Tulsa, OK, USA). Results were significant at *P*<0.05.

## Results

### Inter-bracket comparisons (Table 1)

**Table 1:** Deflection forces (cN) comparison of the different brackets with the use of NC NiTi, RC NiTi, TC NiTi, EC NiTi, and FRP

Deflection (mm)	Polycrystalline (PC)	Metal-insert Polycrystalline (MI-PC)	Monocrystalline (MC)	p
	Force (cN)	Force (cN)	Force (cN)	
	Mean (S.D.)	Mean (S.D.)	Mean (S.D.)	
**Non-coated NiTi (NC)**				
3.0	405.69 (5.66)^A^	390.33 (18.67)^A^	460.48 (27.92)^B^	0.000000*
2.0	268.38 (5.93)^A^	280.20 (9.80)^A^	259.80 (14.97)^B^	0.001104*
1.0	176.29 (19.38)	174.99 (20.22)	195.09 (19.88)	0.055872
0.5	52.75 (30.05)	56.55 (25.37)	61.98 (28.51)	0.779911
**Rhodium coated NiTi (RC)**				
3.0	428.49 (23.86)^A^	408.60 (37.42)^A^	465.88 (23.59)^B^	0.000549*
2.0	308.59 (34.47)	303.04 (39.63)	277.31 (23.89)	0.100181
1.0	187.16 (13.70)^A,B^	173.20 (15.54)^A^	198.86 (12.74)^B^	0.001489*
0.5	24.42 (15.40)^A^	52.91 (27.38)^B^	66.52 (26.48)^B^	0.001647*
**Teflon coated NiTi (TC)**				
3.0	403.73 (25.46)^A^	392.90 (6.46)^A^	434.05 (27.23)^B^	0.000739*
2.0	279.29 (19.52)^A^	287.67 (15.39)^A^	256.09 (16.68)^B^	0.001042*
1.0	128.95 (48.39)^A^	195.05 (19.13)^B^	159.52 (31.83)^A,B^	0.001125*
0.5	41.83 (33.54)^A^	82.99 (27.76)^B^	27.14 (32.50)^A^	0.001356*
**Epoxy coated NiTi (EC)**				
3.0	297.45 (16.03)^A,B^	292.07 (19.28) A	321.19 (30.29)^B^	0.018297*
2.0	131.72 (13.12)	125.11 (5.67)	122.27 (9.55)	0.110971
1.0	122.60 (18.59)	128.67 (14.37)	126.00 (9.64)	0.654518
0.5	32.55 (17.58)	29.04 (27.58)	34.96 (24.44)	0.853368
**Fiber-reinforced polymer (FRP)**				
3.0	214.24 (32.77)^A,B^	194.96 (21.66)^A^	240.87 (31.98)^B^	0.048559*
2.0	29.90 (15.63)^A^	28.30 (18.15)^A^	31.80 (7.36)^A^	0.916206
1.0	14.21 (8.08)^A^	12.98 (13.67)^A^	7.84 (9.54)^A^	0.562287
0.5	0.16 (0.88)^A^	1.36 (6.71)^A^	0.53 (4.17)^A^	0.772254

*Statistically significant at P<0.05

Different letters indicate statistically significant differences (Tukey tests)

The NC NiTi archwire, at 3 mm unloading, presented the statistically highest force in MC brackets. However, at 2 mm unloading, the NC NiTi archwire presented the statistically lowest force in the same brackets (Table 1).

The RC NiTi archwire**,** at 3, 1 and 0.5 mm unloading, presented the highest force in MC brackets (Table 1).

The TC NiTi archwire, at 3 mm unloading, in MC brackets, and at 0.5 mm unloading, in MI-PC bracket, presented the statistically highest force. At 2 mm unloading, in MC brackets, it showed the lowest force. At 1 mm unloading, in MI-PC brackets, it showed the highest force (Table 1).

The EC NiTi archwire, at 3 mm unloading, presented the statistically highest force in MC brackets (Table 1).

The FRP archwire, at 3 mm unloading, presented statistically higher forces in MC brackets. From this point, this archwire had a plastic deformation (crack), producing extremely low forces, near zero, meaning that the archwire stopped exerting force (Table 1).

Inter-archwire comparisons (Table 2)

**Table 2:** Deflection forces (cN) comparison of the different archwires inserted in MI-PC, PC and MC brackets

Deflection (mm)	Non-coated (NC)	Rhodium coated (RC)	Teflon coated (TC)	Epoxy coated (EC)	Fiber-reinforced polymer (FRP)	p
	Force (cN)	Force (cN)	Force (cN)	Force (cN)	Force (cN)	
	Mean (S.D.)	Mean (S.D.)	Mean (S.D.)	Mean (S.D.)	Mean (S.D.)	
**Metal-insert Polycrystalline ceramic (MI-PC)**						
3.0	405.69 (5.66)^A^	428.49(23.89)^A^	403.73 (25.46)^A^	297.45 (16.03)^C^	214.24 (32.77)^B^	0.000000*
2.0	268.38 (5.93)^A^	308.59 (34.47)^D^	279.29 (19.52)^A^	131.72 (13.12)^C^	29.90 (15.63)^B^	0.000000*
1.0	176.29(19.38)^B^	187.16 (13.70)^B^	128.95 (48.39)^A^	122.60 (18.59)^A^	14.21 (8.08)^C^	0.000000*
0.5	52.75 (30.05)^A^	24.42 (15.40)^A,B^	41.83 (33.54)^A^	32.55 (17.58)^A,B^	0.16 (0.88)^B^	0.001637*
**Polycrystalline ceramic (PC)**						
3.0	390.33 (18.67)^A^	408.60 (37.42)^A^	392.90 (6.46)^A^	292.07 (19.28)^C^	194.96 (21.66)^B^	0.000000*
2.0	280.20 (9.80)^A^	303.04 (39.63)^A^	287.67 (15.39)^A^	125.11 (5.67)^C^	28.30 (18.15)^B^	0.000000*
1.0	174.99 (20.22)^A,B^	173.20 (15.54)^A^	195.05 (19.13)^B^	128.67 (14.37)^D^	12.98 (13.67)^C^	0.000000*
0.5	56.55 (25.37)^A,B^	52.91 (27.38)^A,B^	82.99 (27.76)^B^	29.04 (27.58)^A,C^	1.36 (6.71)^C^	0.000002*
**Monocrystalline ceramic (MC)**						
3.0	460.48 (27.92)^A^	465.88 (23.59)^A^	434.05 (27.23)^A^	321.19 (30.29)^C^	240.87 (31.98)^B^	0.000000*
2.0	259.80 (14.97)^A,B^	277.31 (23.89)^B^	256.09 (16.68)^A^	122.27 (9.55)^D^	31.80 (7.36)^C^	0.000000*
1.0	195.09 (19.88)^A^	198.86 (12.74)^A^	159.52 (31.83)^D^	126.00 (9.64)^C^	7.84 (9.54)^B^	0.000000*
0.5	61.98 (32.02)^B^	66.52 (26.48)^B^	27.14 (32.50)^A^	34.96 (24.44)^A,B^	0.53 (4.17)^A^	0.000080*

*Statistically significant at P<0.05

Different letters indicate statistically significant differences (Tukey tests)

The MI-PC bracket presented the highest forces with RC NiTi and NC NiTi archwires for all deflections evaluated (Table 2).

The PC bracket presented the highest forces with RC NiTi and TC NiTi archwires for all deflections evaluated (Table 2).

The MC bracket presented the highest forces with RC NiTi and NC NiTi archwires for all deflections evaluated (Table 2). For all bracket types, the FRP archwire presented the significantly lowest forces for all amounts of unloading.

## Discussion

### Sample and methodology

By presenting fewer variables than clinical tests, *in vitro* comparisons between different brackets and archwires, as performed in this research, present smaller discrepancy of individual responses and more fair and reliable results^19^.

Although the elastic deflection test in the three-point machine is widely used by several authors^3^ ^,^ ^8^ ^,^ ^13^, this research employed a clinical simulation device, as reported by other authors^4^ ^,^ ^9^ ^,^ ^18^, including variables, such as brackets and elastomeric ligatures to best reproduce the clinical environment^1^.

We used super slick elastomeric modules able to generate significantly less static frictional force at the module/archwire interface than regular modules when tied normally.

### Interbracket comparisons

Overall, the brackets presented the following decreasing force ranking: MC, PC and MI-PC, with small variations according to the amount of unloading. Significant interbracket differences tended to occur with large deflections. This happened because the frictional force of the PC brackets is greater, because of its rough surface^21^. Furthermore, the chemical characteristic of alumina on the ceramic surface may cause adherence on the archwire surface. This may generate a high friction and reduce the orthodontic force from 12% to 60%^17^. Because of these problems, a metal-insert has been developed in order to reduce the friction force generated by the ceramic brackets, which is the case of the MI-PC. Development of polycrystalline had already reduced the high friction forces of the ceramic brackets, but the forces were still higher than metal brackets. Therefore, the incorporation of a metal-insert reduced even further the forces generated by these esthetic brackets, decreasing this disadvantageous characteristic^6^.

The highest forces generated by the MC bracket may be related to the bracket composition, which is produced by casting of aluminum oxide particles at very high temperature, followed by controlled cooling, in order to avoid failures in the crystallization. Because of high resistance and a more polished surface, it produces less friction in its insert when combined with orthodontic archwires, releasing higher forces during unloading. Polycrystalline ceramic brackets are produced by agglutinating aluminum oxide particles at lower temperatures, resulting in a rough surface, with greater attrition coefficient and more susceptibility to fracture.

Previous studies have shown that MI-PC bracket produces less friction forces than PC brackets, resulting in higher forces during unloading^6^. However, this should not be extrapolated to this study because of the difference in archwire material composition. Only metal archwires (noncoated) were used in those studies, but ours used only coated archwires.

It is suggested that small variations in the amount of unloading produced by different types of brackets are closely related to the friction caused by different ceramic bracket and esthetic archwire material combinations, because these materials have a more rough surface than metal noncoated archwires^1^ ^,^ ^9^ ^,^ ^18^ ^,^ ^20^. This may be illustrated in our results during the tests performed with the MI-PC bracket combined with TC NiTi archwire, which has only esthetic coating on their labial surface. Overall, the highest forces on unloading (2.0 mm, 1.0 mm and 0.5 mm) were presented by the MI-PC bracket.

### Inter-archwire comparisons

The overall decreasing force ranking of the archwires was: RC NiTi, NC NiTi, TC NiTi, EC NiTi and FRP. TC NiTi exhibited higher forces than NC NiTi only in the PC brackets. Significant inter-archwire differences occurred with several amounts of deflections.

The RC NiTi is plated with Rhodium, a noble, ductile silver white colored metal. It is suggested that its esthetic cover layer with low friction characteristics, should contribute to release higher forces during unloading, as it did. Accurate data about the manufacturing process and conditions of this archwire were not available by the manufacturer. Nevertheless, in a recent study, RC archwires showed the highest surface roughness, greater elasticity and strength during activation, but not higher forces on unloading^20^.

The use of a NC NiTi archwire was necessary as a parameter to be followed by the other archwires, since, with exception of the FRP archwire, all the other NiTi archwires were coated with some type of esthetic material. The forces generated by this archwire were only smaller than the RC NiTi, in every situation, and to the TC NiTi in PC brackets.

The TC archwire generated the third greatest force possibly caused by the surface of the esthetic coating, influencing the resistance to sliding during force measurement at unloading^12^. Recent studies have shown that coated archwires may now be able to generate forces similar to the NC archwire, particularly when only the labial surface of the archwire is coated, such as this one^18^.

Among all tested archwires, with and without coating, EC archwire showed the lowest unloading force values for all deflections. This may be related to increased friction arising from its coating material, since the greater the friction at the archwire/bracket interface, the lower the force generated during unloading, because friction consumes part of the accumulated initial force during archwire activation. Only from the moment that it exceeds the static friction, the archwire will actually express its stored energy. This result reinforces other findings that found lower force generated by EC archwires^1^ ^,^ ^9^ ^,^ ^18^. The differences observed between teflon and epoxy coated archwires occur probably because teflon coating is only performed in the labial aspect of the archwire.

**Figure 5:**
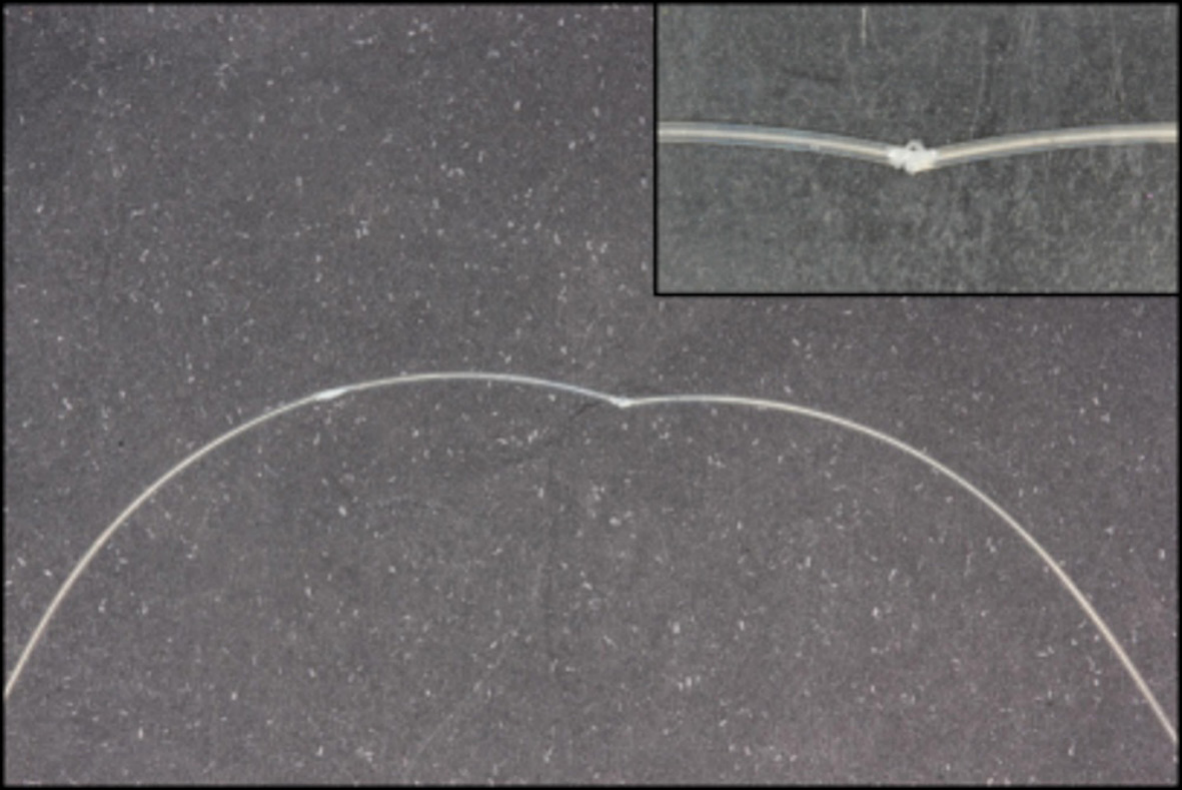
Crack generated on the FRP archwire during deflection (Optis/TP Orthodontics®; La Porte, Indiana, USA)

In this research, the purely esthetic archwire FRP presented the lowest force values for all deflections. After 3 mm of deflection, the archwire had permanent deformation (crack), meaning that the archwire stopped exerting force (Figure 5). Cracking is defined as a region of ultrafine cracks in the resin phase leading to the appearance of a white band^25^. We noticed by a significant drop in force values. Even with the cracking, the archwires still exert some force, but they are much lower than those without cracking^7^.

The FRP archwire is manufactured with translucent composite material comprised of a poly(methyl methacrylate) matrix (PMMA) and glass fiber for reinforcement to obtain a final product not only esthetic, but also able to reproduce the mechanical properties of the coated NiTi archwires. It should display satisfactory springback to provide an adequate tooth movement. In other words, the archwire should return to its original format after tied to the teeth.

During its manufacturing process, the fiber content that comprises the structure, influences force variability and rigidity^15^. The size and amount of fiber filaments determine changes in rigidity of the archwire, also altering the elasticity modulus and the elastic limit^15^. Thus, it is suggested that the 0.016-in FRP archwire evaluated in this research has an internal fiber configuration unable to withstanding 3 mm of elastic deflection and keep their original shape without losing the stiffness and elasticity.

A similar result was obtained by Spendlove, et al.^28^ (2015) who found fracture of the archwires and decrease of the force released in 2 mm of deflection. Likewise, Chang, et al.^7^ (2014) observed microcracks in the structure of 30% of the esthetic archwire samples, stored in water for 30 days, warning for its limited clinical employment, as a viable clinical orthodontic archwire. Also, Huang, et al.^11^ (2003) tested a new fiber glass archwire, which similarly fractured under a deflection slightly larger than 2 mm.

The clinical applicability of these FRP archwires may be limited as they are unable to sustain deflections of 2 mm without experiencing cracking and loss of force delivery. Studies performing microscopic analysis of failures are interesting to investigate the cause of archwire crack, and thus associate them with the falling load values during unloading.

### Clinical considerations

The results of this study do not allow a thorough comparison with previous studies since aspects, such as archwire size, deflection values, brackets and elastomeric rings and temperature are variables that need to be considered^9^ ^,^ ^13^ ^-^ ^15^ ^,^ ^18^.

The optimal deflection occurs at clinically useful displacements between 1 and 2.5 mm. These are the movements that predominate during leveling and aligning with low dimension archwires. This unloading region is the force value most likely to be applied in the clinical situation as soon as some movement of the teeth has occurred within the periodontal ligament^10^.

Reduction in the internal dimensions of NiTi archwire, to compensate for the coating thickness, seems to be responsible for the major changes in mechanical properties of the esthetic archwires, particularly in elastic deflection forces, as observed^18^.

The esthetic archwires employed in this study presented mostly deflection forces comparable to those obtained by NC NiTi archwires. Special attention should be given to the FRP archwire that, despite being highly esthetic, presented a permanent crack with 3 mm of deflection. This means that when the glass fiber archwire is employed in moderate to severe crowding, it may undergo permanent bending, interrupting tooth movement.

Since there are few published studies on the mechanical properties of esthetic archwires, additional studies need to be conducted, so that these mechanical properties are consistent with the desired force levels to induce tooth movement.

Furthermore, additional investigation is necessary to clarify whether the differences observed above reflect the actual influence of the coating material or if they are influenced by the coating manufacturing process.

## Conclusions

- Overall, MC brackets presented the best results, because they produced the highest forces during unloading (lower friction), followed by PC brackets and, finally, by MI-PC brackets, with small variations according to the amount of unloading;
- Overall, RC NiTi archwire presented the best results, because it produced the highest forces during unloading (lower friction), followed by NC NiTi, TC NiTi, EC NiTi and FRP archwires. Combinations of these archwires with the brackets will result in a proportional ranking;
- The FRP archwire presented plastic deformation at 3 mm of deflection and produced extremely low forces at 2; 1 and 0.5 mm of deflection, not comparable with the mean forces generated by the other tested archwires;
- Esthetic brackets and archwires, when used together, can exhibit very different patterns of forces because of the bracket composition and type of archwire coating.
